# Lack of Intestinal Epithelial Atg7 Affects Paneth Cell Granule Formation but Does Not Compromise Immune Homeostasis in the Gut

**DOI:** 10.1155/2012/278059

**Published:** 2012-01-12

**Authors:** Nadine Wittkopf, Claudia Günther, Eva Martini, Maximilian Waldner, Kerstin U. Amann, Markus F. Neurath, Christoph Becker

**Affiliations:** ^1^Medical Clinic 1, Friedrich-Alexander-University Erlangen-Nuremberg, 91054 Erlangen, Germany; ^2^Department of Nephropathology, Friedrich-Alexander-University Erlangen-Nuremberg, 91054 Erlangen, Germany

## Abstract

Genetic polymorphisms of autophagy-related genes have been associated with an increased risk to develop inflammatory bowel disease (IBD). Autophagy is an elementary process participating in several cellular events such as cellular clearance and nonapoptotic programmed cell death. Furthermore, autophagy may be involved in intestinal immune homeostasis due to its participation in the digestion of intracellular pathogens and in antigen presentation. In the present study, the role of autophagy in the intestinal epithelial layer was investigated. The intestinal epithelium is essential to maintain gut homeostasis, and defects within this barrier have been associated with the pathogenesis of IBD. Therefore, mice with intestinal epithelial deletion of Atg7 were generated and investigated in different mouse models. Knockout mice showed reduced size of granules and decreased levels of lysozyme in Paneth cells. However, this was dispensable for gut immune homeostasis and had no effect on susceptibility in mouse models of experimentally induced colitis.

## 1. Introduction

Inflammatory bowel disease (IBD) with its two major subtypes Crohn's disease (CD) and ulcerative colitis (UC) is a chronic relapsing inflammatory disorder of the gastrointestinal tract approximately affecting 400 in 100,000 of the adult population in western countries [1]. Patients suffer from varying symptoms including abdominal pain, diarrhoea, rectal bleeding, weight loss, and lethargy. The pathogenesis of IBD is still under investigation but several factors have been shown to play a role in the aetiology of IBD including environmental triggers, genetic factors, bacterial flora, and an overreactive immune response. It is a generally accepted theory that the disease arises due to a dysfunctional interaction between the mucosal immune system and the bacterial microflora in genetic susceptible humans. In healthy individuals, the internal mucosal tissue is separated from the intestinal lumen including food antigens and bacteria by the intestinal epithelial barrier playing a crucial role for gut homeostasis. Disturbance of the epithelial barrier function has been demonstrated to potentially induce intestinal inflammation [2, 3].

Among the identified genetic factors providing an increased risk for the development of IBD, autophagy-related genes such as ATG16L1, IRGM1, and LRRK2 suggest the implication of autophagy dysregulation in the pathogenesis of IBD [4–6]. Autophagy has been demonstrated to be elementary for cellular homeostasis as it is involved in cellular clearance. During the autophagy process, cellular constituents such as aged and damaged organelles or proteins are enveloped by membranes and delivered to lysosomal vesicles for degradation. Furthermore, autophagy is involved in other cellular processes such as development, cellular differentiation, aging, and nonapoptotic programmed cell death [7–9]. It also participates in the clearance of apoptotic bodies of dying cells to prevent tissue inflammation further providing possible implications of autophagy in the pathogenesis of intestinal inflammatory diseases [10]. The association between IBD and autophagy dysregulation is further supported by the role of autophagy in immunity because autophagy participates in the digestion of intracellular pathogens and is involved in antigen presentation [11]. The importance of autophagy has been underlined by the generation of autophagy knockout mice. Mice with a general deletion of the autophagy proteins Atg5, Atg7, or Atg16L1 die within few hours after birth due to the neonatal starvation period [12–14]. Atg7 is an enzyme playing a central role in the elongation of autophagy vesicle membranes. Recent data have shown that liver cell-specific Atg7 deficiency caused hepatomegaly due to accumulation of abnormal organelles and cell swelling [12] while Atg7 deletion in Purkinje cells led to degeneration of axon terminals followed by mouse behavioural deficits [15]. Furthermore, mice with defective Atg16L1 protein in the haematopoietic system were highly susceptible to dextran sodium sulphate- (DSS-) induced colitis, again indicating the crucial role of autophagy in the pathogenesis of IBD [14].

Because of the important role of autophagy in many cellular processes, the association between inflammatory bowel disease and autophagy, the critical involvement of the intestinal epithelial cell (IEC) layer in the pathogenesis of IBD and its special position at the interface between inside and outsides, the role of autophagy in intestinal epithelial cells was investigated. Here, we demonstrated by the analysis of IEC-specific conditional knockout mice that Atg7 deficiency alters the granular morphology of Paneth cells but that Atg7 is generally dispensable for gut homeostasis.

## 2. Material and Methods

### 2.1. Mice

Mice carrying a loxP-flanked Atg7 allele (Atg7^fl^) were kindly provided by Komatsu and Tanaka [12]. C57BL/6 mice carrying the sequence for the Cre recombinase under control of the villin promoter (Villin-Cre mice) were described earlier [16]. Atg7^fl^ mice were crossbred with Villin-Cre mice to generate intestinal epithelial-specific Atg7 knockout mice (Atg7^IEC-KO^). Mice were kept in individually ventilated cages.

### 2.2. Experimental Model of Intestinal Inflammation

Experimental colitis was induced by challenging mice with dextran sodium sulphate (DSS, MP Biomedicals). 3% DSS were dissolved in sterile drinking water and the solution was continuously applied to the mice as drinking water. DSS solution was exchanged every other day. Development of colitis was monitored by weighing the mice and by regular colonoscopy as previously described [17]. The extent of inflammation was scored as previously performed [17, 18].

### 2.3. Histological Examination

Freshly isolated tissues were either instantly frozen in liquid nitrogen or fixed in 4% formalin and then paraffin embedded. Sections of paraffin embedded tissues were stained with H & E or combined staining methods with alcian blue, PAS, Elastica, and van Gieson to visualize tissue structures. Immunohistochemical analysis of cryosections was performed using Anti-CD11c antibodies (BD Pharmingen), Anti-lysozyme antibodies (Dianova), and Anti-myeloperoxidase antibodies (Abcam) as primary antibodies, biotinylated secondary antibodies (Dianova) and the TSA Cy3 system (PerkinElmer) as recommended by the manufacturer. Apoptotic cells were detected using In situ Cell Death Detection Kit Fluorescein (Roche) for TdT-mediated dUTP nick end labelling (TUNEL) according to manufacture recommendations. Bacteria were detected by fluorescence in situ hybridization (FISH) of bacterial RNA as previously described [19]. Nuclei were counterstained with Hoechst 3342 (Invitrogen). Immunofluorescent tissue slices were analysed using a fluorescence microscope (Olympus). For analysis of tissues by electron microscopy, tissues were fixed using glutaraldehyde and further embedded in Epon Araldite. Ultrathin sections were analysed using an electron microscope (Zeiss).

### 2.4. IEC Isolation and Western Blotting

Intestinal epithelial cells were isolated by carefully removing the whole intestine from the mouse corpus, inversion of the intestine, washing in phosphate-buffered saline to clean intestine from feces, and incubating the tissue in prewarmed isolation solution containing HBSS (PAA), 1 mM EGTA (Sigma), 2 mM EDTA (Sigma), and 10% FCS (PAA) for 15 minutes at 37°C. Subsequently, isolated cells were pelleted at 1200 rpm and 4°C for 5 minutes and washed twice with 1x PBS and repeated centrifugation. Proteins were extracted using the mammalian protein extraction reagent (Thermo Scientific) containing protease and phosphatase inhibitor tablets (Complete Mini Protease Inhibitor Cocktail Tablets and PhosStop Phosphatase Inhibitor Cocktail Tablets, Roche). Proteins were separated according to their molecular weight by SDS polyacrylamide gel electrophoresis and subsequent transfer to Protran nitrocellulose transfer membrane (Whatmann). Membranes were blocked in Roti-Block (Roth) and probed with Anti-Atg7-CT antibody (AnaSpec) or Anti-LC3B-antibody (Cell Signaling) over night at 4°C with gentle shaking followed by incubation with secondary HRP-linked Anti-Rabbit antibody (Cell Signaling). Incubating membranes with HRP-linked Anti-Actin antibody (Santa Cruz Biotechnology) for 1 hour at room temperature served as an internal control. For detection of protein bands, Western Lightning Plus-ECL (PerkinElmer) was used according to manufacture recommendations.

### 2.5. Transcription Analysis

Total RNA was extracted from tissues using an RNA isolation Kit (Nucleo Spin RNA II, Macherey Nagel) and cDNA was generated by reverse transcription using the iScript cDNA Synthesis Kit (Bio-Rad). cDNA samples were mixed with SsoFast EvaGreen (Bio-Rad) and specific QuantiTect Primer assays (Qiagen) and analysed by real-time PCR. *Hprt* was used as an internal control.

### 2.6. Statistical Analysis

Statistical analysis was performed using Student's *t*-test. Double asterisks indicate significant differences (*P* < 0.01). n.s. = nonsignificant differences (*P* > 0.05).

## 3. Results and Discussion

**Figure 1:**
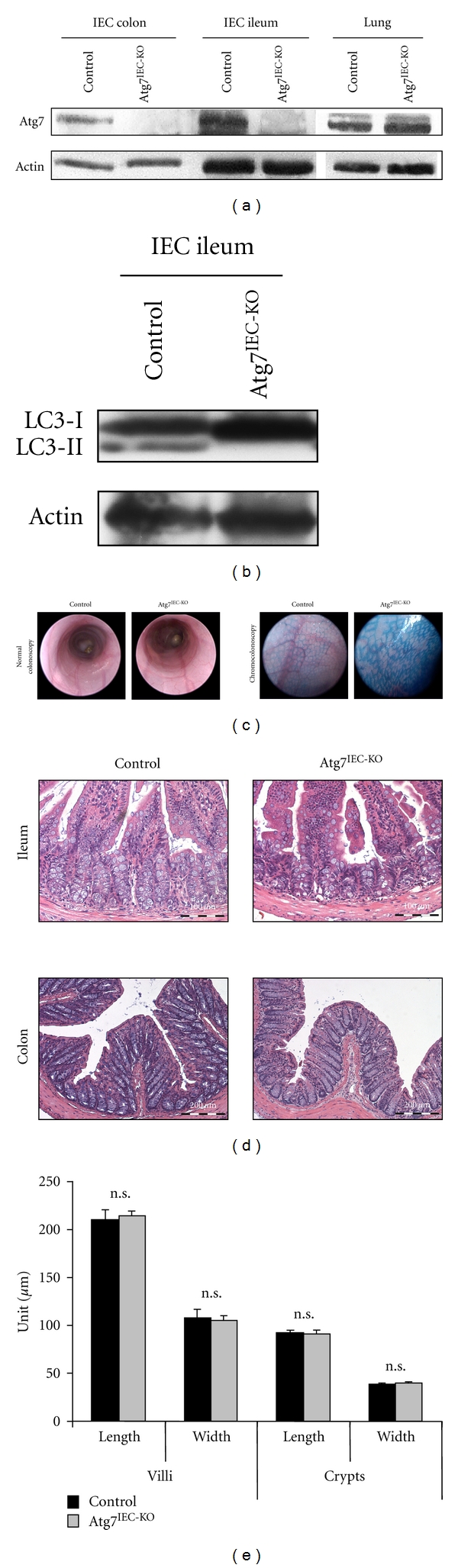
Gut characteristics in unchallenged control and Atg7^IEC-KO^ mice. (a) Western blot of proteins derived from unchallenged control and Atg7^IEC-KO^ mice demonstrating lack of Atg7 in isolated intestinal epithelial cells of colon and ileum from Atg7^IEC-KO^ mice. Other tissues of Atg7^IEC-KO^ mice display normal Atg7 expression (lung is shown as an example). Actin serves as an internal control. (b) Western blot of proteins extracted from isolated IECs of unchallenged control and Atg7^IEC-KO^ mice demonstrating deficient autophagy in Atg7-deficient IECs (indicated by lack of the LC3-II form). Actin serves as an internal control. (c) Representative pictures from colonoscopic video analysis using normal colonoscopy (left) and chromocolonoscopy with methylene blue (right) to visualize crypt structures. (d) Representative pictures of paraffin-embedded ileum and colon cross sections stained with H & E. (e) Statistical analysis of distal ileum villi and crypts. Data show mean values of length and width + SEM (*n* = 13 control villi, *n* = 24 Atg7^IEC-KO^ villi, *n* = 24 control crypts, *n* = 27 Atg7^IEC-KO^ crypts).

To investigate the role of autophagy in the intestinal epithelial cell layer, we crossbred Villin-Cre mice with mice carrying loxP-flanked Atg7 alleles to generate conditional knockout mice (Atg7^IEC-KO^ mice). Atg7^IEC-KO^ mice showed an IEC-specific deletion of the autophagy protein Atg7 (Figure 1(a)), which is essential for the elongation of autophagy vesicles. During the autophagy process, the cytosolic LC3-I is converted to the lipidated LC3-II by an ubiquitin-like conjugation system involving Atg7. LC3-II is widely accepted as a marker for activated autophagy [20, 21]. In control intestinal epithelial cells, both LC3-I and LC3-II were detected by western blotting. In contrast, only the LC3-I form was observed at an increased level in Atg7-deficient IECs indicating impaired autophagy in these cells (Figure 1(b)). IEC specific Atg7 conditional knockout mice were born healthy and fertile and did not reveal an overt phenotype compared to control littermates. To examine the influence of Atg7 deficiency in IECs on gut homeostasis, the colon of conditional knockout mice and control mice was analysed by colonoscopy. Despite the supposed role of autophagy in the pathogenesis of inflammatory bowel disease in humans, no macroscopic differences were detected indicating that Atg7 deficiency is not associated with spontaneous gut inflammation (Figure 1(c)). Furthermore, chromocolonoscopy using methylene blue demonstrated normal crypt morphology within the colon (Figure 1(c)). H & E staining of colonic cross sections further confirmed the lack of structural alterations in Atg7^IEC-KO^ mice (Figure 1(d)); a finding that was underlined by morphometric analysis, demonstrating comparable general structures of colon and ileum such as length and width of villi and crypts (Figure 1(e)).

**Figure 2:**
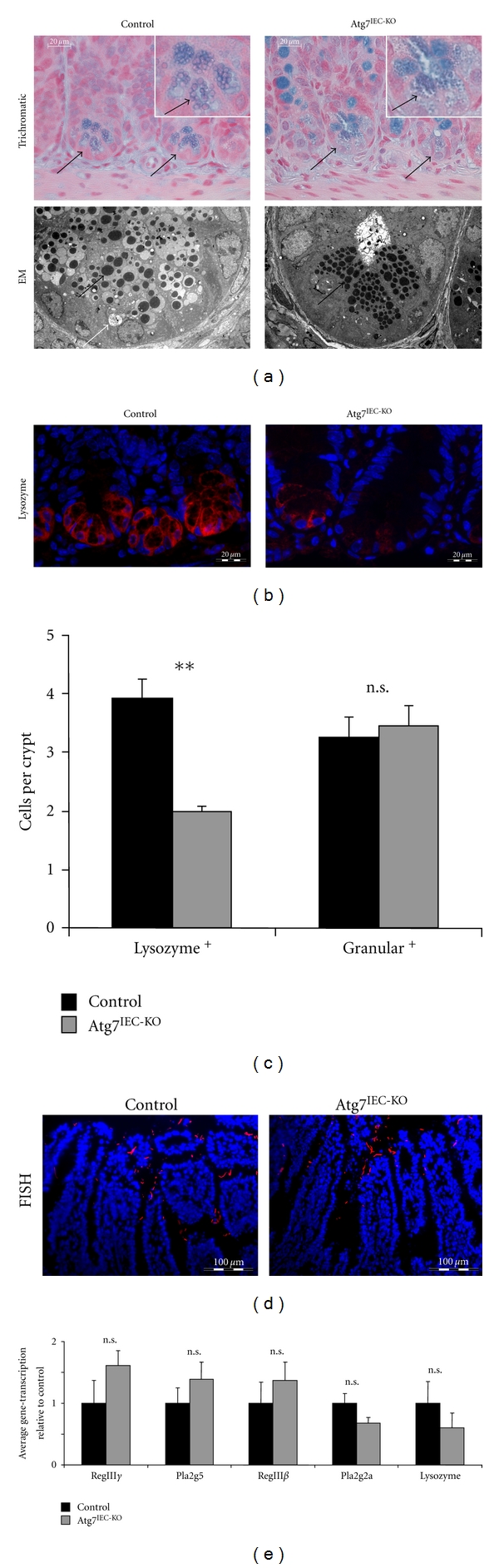
Paneth cell function in unchallenged control and Atg7^IEC-KO^ mice. (a) Histological analysis of Paneth cells by trichromatic staining with alcian blue, PAS, and Elastica van Gieson (top) and by electron microscopy (EM, bottom, 2156x magnification) demonstrates smaller size of granules (black arrows) in Atg7-deficient Paneth cells compared to control Paneth cells. Large autophagosomes (white arrow) were detectable in electron microscopic pictures of IECs derived from control mice but not of IECs derived from Atg7^IEC-KO^ mice. (b) Immunofluorescent staining of distal small intestine for lysozyme (red) reveals decreased levels of lysozyme in Atg7 deficient Paneth cells. Nuclei are shown in blue. (c) Statistical analysis of the number of cells containing granules (granular+) or lysozyme (lysozyme+) at the base of crypts in the distal small intestine of unchallenged control and Atg7^IEC-KO^ mice (*n* = 187 control crypts and *n* = 140 Atg7^IEC-KO^ crypts for analysing granular+ cells, *n* = 75 control crypts and *n* = 43 Atg7^IEC-KO^ crypts for analysing lysozyme+ cells). (d) Detection of bacteria (red) in the distal small intestine of unchallenged control and Atg7^IEC-KO^ mice by fluorescence in situ hybridization (FISH). Nuclei are shown in blue. (e) Quantitative analysis of the transcription of antimicrobial peptide genes in the distal small intestine of unchallenged control and Atg7^IEC-KO^ mice. Data show mean values + SEM (*n* = 5 control mice, *n* = 4 Atg7^IEC-KO^ mice).

Recent studies have demonstrated alterations in Paneth cells of autophagy-deficient mice [22]. Interestingly, Paneth cells of Atg7^IEC-KO^ mice investigated in the current study showed a different morphology compared to Paneth cells of control mice as demonstrated by combined staining of distal small intestine paraffin sections with alcian blue, PAS, Elastica, and van Gieson and by electron microscopy (Figure 2(a)). Accordingly, morphological alterations in Atg7-deficient Paneth cells were indicated by the appearance of more but smaller vesicles compared to Paneth cells in control mice (Figure 2(a), black arrows), suggesting that Atg7 deficiency led to irregularities in granule formation. Paneth cell granules are storage vesicles containing, for example, CD95 ligand, TNF-*α*, and antibacterial substances such as lysozyme, secretory phospholipase A2, RegIII*γ*, and IgA [23]. Importantly, Paneth cell granules are secreted into the gut lumen and participate in innate immune defence. Interestingly, immunofluorescence staining revealed reduction of lysozyme, a marker of Paneth cells, in the small intestine of unchallenged Atg7^IEC-KO^ mice compared to control (Figure 2(b)). A careful statistical analysis of the number of cells at the base of the crypt containing either granules or lysozyme demonstrated that although the number of lysozyme positive cells was decreased, the number of cells containing granules was comparable between Atg7^IEC-KO^ mice and control mice, suggesting that Atg7 deficiency affects the storage and secretion of lysozyme rather than the development or survival of Paneth cells (Figure 2(c)). This was further underlined by quantitative analysis of transcription levels of antimicrobial peptides (AMPs) secreted by Paneth cells demonstrating comparable transcription levels of RegIII*γ*, RegIII*β*, Pla2g2a, and Pla2g5 in distal small intestine of unchallenged control and Atg7^IEC-KO^ mice (Figure 2(e)). Furthermore, although diminished levels of lysozyme were detectable in ileal cross sections by immunofluorescence analysis, gene transcription of lysozyme in the distal small intestine of Atg7^IEC-KO^ mice was not significantly different from control mice. Our data are in agreement with data from Cadwell et al., demonstrating altered granular morphology in Atg16L1 and Atg7-deficient Paneth cells [24]. However, while Cadwell et al. reported decreased amounts of granules and diffuse lysozyme staining in Paneth cells of both mice, in contrast, we observed increased numbers and smaller sizes of Paneth cell granules and decreased lysozyme staining. Disturbed lysozyme secretion by Paneth cells suggested decreased antimicrobial defence and alterations in the microbial flora in the intestine of Atg7^IEC-KO^ mice. However, no alterations in the amount of bacteria or the attachment of bacteria to the intestinal epithelial layer were detected (Figure 2(d)). Further studies should investigate whether diminished secretion of the AMP lysozyme by Atg7 deficient Paneth cells influences the composition of the bacterial microflora. In a recent study, Cadwell et al. detected no increased susceptibility of Atg16L1-hypomorphic mice to oral infection with *Listeria monocytogenes*. In agreement with this finding, we found that clearance of orally applied *Citrobacter rodentium*—a commonly used mouse gram negative pathogen mimicking human infectious colitis—was also not affected by IEC Atg7 deficiency (data not shown). Collectively, this implies that Atg7 deficiency and decreased lysozyme in Paneth cells do not affect the attachment of bacteria to IECs and the susceptibility to infections with gram negative bacteria.

**Figure 3:**
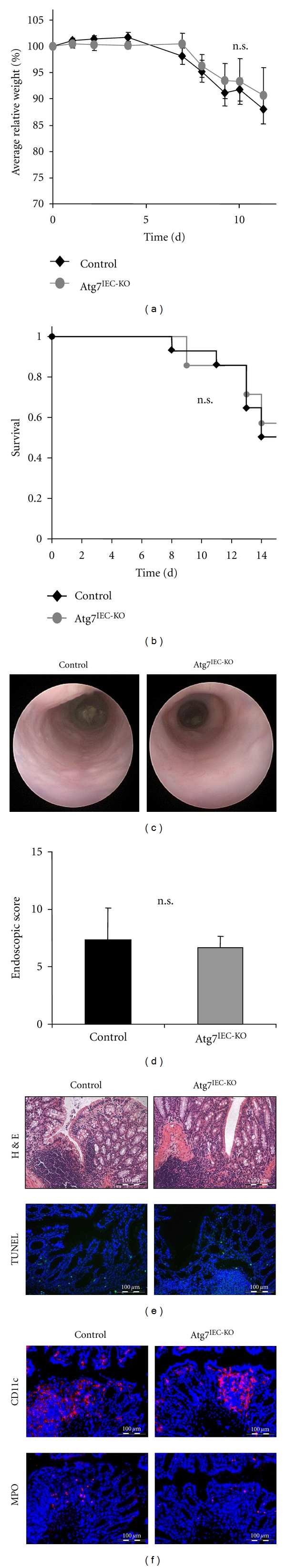
Experimentally induced colitis in control and Atg7^IEC-KO^ mice. Control and Atg7^IEC-KO^ mice were continuously challenged with 3% DSS in the drinking water. Illustrated data are representative (*n* = 4 independent experiments). (a) Average weight dynamics were calculated from mouse body weights relative to day 0. Data show mean values ± SEM (*n* = 14 control mice, *n* = 7 Atg7^IEC-KO^ mice). (b) Survival analysis. (c) Colonoscopic pictures of control and Atg7^IEC-KO^ mice 14 days after beginning of DSS treatment. (d) Scoring of the extent of colitis. Data show mean values + SEM (*n* = 3 mice in each group). (e) Histological analysis of the distal part of the colon from control and Atg7^IEC-KO^ mice, treated with DSS for 14 days, by H & E staining (upper row) and TUNEL (bottom row). (f) Infiltration of CD11c+ cells (red, upper row) and MPO+ cells (red, bottom row) into the colon of DSS-treated control and Atg7^IEC-KO^ mice was detected by immunohistochemical analysis. Nuclei are shown in blue.

Since previous studies had demonstrated the association between autophagy dysregulation and the pathogenesis of IBD [4–6] and also detected decreased secretion of Paneth cell AMPs in the gut of Crohn's disease patients [25, 26], we reasoned that IEC-specific Atg7-deficient mice could be more susceptible to experimentally induced intestinal inflammation. In order to investigate whether deficiency of Atg7 in IECs might modulate intestinal homeostasis under disease conditions, colonic inflammation was induced using dextran sodium sulphate, a commonly used experimental colitis model in mice. Atg7^IEC-KO^ and control mice were continuously treated with 3% DSS in the drinking water. As demonstrated by monitoring mouse body weight changes as an indicator for the general mouse health and by survival analysis, all mice responded comparable to DSS treatment (Figures 3(a) and 3(b)). Development of colitis was followed using colonoscopic video analysis and inflammation scoring. Atg7 deficiency did not affect severity of DSS-induced colitis as demonstrated by comparable signs of inflammation such as granularity of the mucosa, fibrin formation, vascular structure, stool loosening, and thickening of the bowel wall (Figures 3(c) and 3(d)). Extent of inflammation in the colon of DSS-treated Atg7^IEC-KO^ mice was further analysed by TUNEL and H & E staining demonstrating similar tissue destruction and infiltration of immune cells in both mice (Figure 3(e)). We also detected comparable infiltration of immune cells such as dendritic cells and granulocytes into the colonic lamina propria of DSS-treated Atg7^IEC-KO^ mice and control littermates by immunofluorescence staining (Figure 3(f)). These data demonstrate that Atg7 in intestinal epithelial cells is not essential to manage DSS-induced colitis.

## 4. Conclusion

In summary, we have analysed intestinal epithelial-specific Atg7-deficient mice and challenged them with mouse models of experimentally induced colitis. Our data suggest that deficiency of the autophagy protein Atg7 selectively affects Paneth cell granule formation while no other intestinal epithelial cell lineage seemed to be affected. This finding is surprising, as many other autophagy-related conditional knockout mice have demonstrated severe phenotypes and impaired homeostasis of the respective organs. For example, loss of Atg7 in the liver led to cell swelling due to accumulation of abnormal organelles in hepatic cells and mutant mice developed hepatomegaly [12]. Neural-cell-specific Atg7 knockout mice also developed a severe phenotype as they had a decreased number of Purkinje cells leading to behavioural deficits [15, 27]. Although Atg7 deletion was demonstrated to result in autophagy deficiency in IECs, we did neither observe cell swelling nor increased cell death suggesting that autophagy is dispensable for homeostasis of the intestinal epithelium in healthy individuals. We propose that no other cells than Paneth cells are affected by Atg7 deficiency as these cell lineages (enterocytes, goblet cell, and enteroendocrine cells) have a shorter lifetime (4-5 days) [28]. In these cells, autophagy might be not essential as an intracellular clearance mechanism as damaged organelles and proteins may not accumulate to toxic concentrations, given the short lifetime of these cells. The susceptibility of Paneth cells to Atg7 deficiency may be based on their longer lifespan and the abundant endoplasmic reticulum (ER). Autophagy defects might lead to impaired turnover of ER resulting in increased endoplasmic reticulum stress. For example, mice deficient for Xbp1, a transcription factor required for ER expansion, have reduced numbers of lysozyme-positive Paneth cells and remaining Paneth cells contained compressed ER demonstrating the crucial role of ER homeostasis for Paneth cell biology [29]. Furthermore, membranes derived from the endoplasmic reticulum are the source of many intracellular membranous bodies suggesting that increased ER stress caused by autophagy deficiency results in impaired formation of granules in Paneth cells of Atg7^IEC-KO^ mice.

Interestingly, we could not detect inflammatory alterations in unchallenged Atg7^IEC-KO^ mice suggesting that Atg7 deficiency in the intestinal epithelium is not sufficient to induce a Crohn's disease like phenotype. This was very surprising, as polymorphisms in autophagy genes have been associated with an increased risk to develop inflammatory bowel disease. Furthermore, other studies have demonstrated that rapid clearance of apoptotic bodies is essential to prevent tissue inflammation [30] and that Atg5 general knockout mice showed a decreased removal of apoptotic cells and increased tissue inflammation [10]. However, the absence of tissue inflammation in unchallenged Atg7^IEC-KO^ mice might be reasoned by a minor role of autophagy in short living tissues such as the intestinal epithelial cell layer and the shedding of dead IECs into the gut lumen being unable to cause inflammation. The functional role of Atg7-mediated autophagy in the pathogenesis of IBD was further analysed in experimental models of colitis. Although Atg7^IEC-KO^ mice displayed Paneth cell granule abnormalities similar to those observed in Crohn's disease patients [22], Atg7^IEC-KO^ mice did not show increased susceptibility towards DSS-induced or* Citrobacter rodentium* induced colitis. However, other environmental triggers such as intestinal infections with *Salmonella typhimurium*—an intracellular pathogen requiring autophagy for its clearance—might be a more promising experimental model and should be analysed for its relevance in Atg7^IEC-KO^ mice. In contrast to our data, Cadwell et al. showed an increased susceptibility of hypomorphic Atg16L1 mice to DSS-induced colitis. However, this effect was dependent on the infection with a certain virus strain [31]. Furthermore, hypomorphic Atg16L1 mice have a defective autophagy in all cell types and therefore, these results are not comparable to our study. Thus, our data show that Atg7 deficiency in the intestinal epithelium alone does not lead to altered responses to DSS treatment indicating that autophagy dysregulation in intestinal immune cells might play a more important role for the pathogenesis of IBD.

In conclusion, these data demonstrate that Atg7 deficiency in IECs affects Paneth cell biology and suggest that Atg7 in intestinal epithelial cells is dispensable for gut homeostasis. Further studies have to investigate why stem cells, although they are long living, seem to be unaffected by Atg7 deficiency and if other environmental triggers render Atg7^IEC-KO^ mice more susceptible to experimentally induced colitis.
